# Neutralization by Metal Ions of the Toxicity of Sodium Selenide

**DOI:** 10.1371/journal.pone.0054353

**Published:** 2013-01-14

**Authors:** Marc Dauplais, Myriam Lazard, Sylvain Blanquet, Pierre Plateau

**Affiliations:** École polytechnique, Laboratoire de Biochimie, CNRS, Palaiseau, France; Auburn University, United States of America

## Abstract

Inert metal-selenide colloids are found in animals. They are believed to afford cross-protection against the toxicities of both metals and selenocompounds. Here, the toxicities of metal salt and sodium selenide mixtures were systematically studied using the death rate of *Saccharomyces cerevisiae* cells as an indicator. In parallel, the abilities of these mixtures to produce colloids were assessed. Studied metal cations could be classified in three groups: (i) metal ions that protect cells against selenium toxicity and form insoluble colloids with selenide (Ag^+^, Cd^2+^, Cu^2+^, Hg^2+^, Pb^2+^ and Zn^2+^), (ii) metal ions which protect cells by producing insoluble metal-selenide complexes and by catalyzing hydrogen selenide oxidation in the presence of dioxygen (Co^2+^ and Ni^2+^) and, finally, (iii) metal ions which do not afford protection and do not interact (Ca^2+^, Mg^2+^, Mn^2+^) or weakly interact (Fe^2+^) with selenide under the assayed conditions. When occurring, the insoluble complexes formed from divalent metal ions and selenide contained equimolar amounts of metal and selenium atoms. With the monovalent silver ion, the complex contained two silver atoms per selenium atom. Next, because selenides are compounds prone to oxidation, the stabilities of the above colloids were evaluated under oxidizing conditions. 5,5'-dithiobis-(2-nitrobenzoic acid) (DTNB), the reduction of which can be optically followed, was used to promote selenide oxidation. Complexes with cadmium, copper, lead, mercury or silver resisted dissolution by DTNB treatment over several hours. With nickel and cobalt, partial oxidation by DTNB occurred. On the other hand, when starting from ZnSe or FeSe complexes, full decompositions were obtained within a few tens of minutes. The above properties possibly explain why ZnSe and FeSe nanoparticles were not detected in animals exposed to selenocompounds.

## Introduction

Besides its essential role in selenoenzymes, selenium, which is highly toxic itself at elevated levels, is implicated in metal detoxification. The metalloid was early shown to protect animals against the toxic effects of a number of metals including cadmium, mercury and silver (for review, see [1]). Reciprocally, selenium toxicity can be antagonized by metal ions [2], [3]. Correlations could be made between the levels of mercury and selenium in either marine mammals [4] or man [5], highlighting that environmental or occupational exposition to mercury increased not only the content of this metal in various organs but at the same time that of selenium. These observations suggested that selenium and mercury engage themselves in innocuous complexes. Indeed, HgSe particles could be early detected in various tissues of wild marine mammals [6]. Ag_2_Se particles were later found in the kidney and the liver of a human patient treated for tooth decay with silver nitrate [7]. Finally, when simultaneously administered to rats, cadmium and selenium were partly recovered in high-molecular-weight (HMW) protein complexes [8]. The formed complexes contained equimolar amounts of selenium and cadmium [9]. In these experiments, selenium was given under the form of sodium selenite. In fact, to obtain HMW Cd-Se complexes in rat plasma, the former conversion of selenite to selenide by erythrocytes is required [9], [10], [11].

Hydrogen selenide in solution (a mixture of H_2_Se, HSe^–^ and Se^2–^, in chemical equilibrium) forms complexes of very low solubility with numerous metal salts [12]. At this stage, several questions on the relationships between selenide and metal detoxifications can be raised. Can all metallic cations react with hydrogen selenide to form insoluble colloids? When they occur, do these colloids afford protection against selenium toxicity? Finally, how stable are the colloids in the presence of biological oxidizing agents such as dioxygen, oxidized glutathione, flavins,…? To answer these questions, we examined the ability of Ca^2+^, Cd^2+^, Co^2+^, Cu^2+^, Fe^2+^, Hg^2+^, Mg^2+^, Mn^2+^, Ni^2+^, Pb^2+^ Zn^2+^ or Ag^+^ cations to produce colloidal precipitates in the presence of sodium selenide. Next, *Saccharomyces cerevisiae* cells, whose sensitivity to selenite/selenide or metals has already been largely explored [13], [14], [15], [16], [17], [18], were exposed to various sodium selenide-metal salt mixtures. Thereafter the survival rate was measured. In a final set of experiments, the stability of the above colloids in the presence of an oxidizing agent was assessed by using 5,5'-dithiobis-(2-nitrobenzoate) (DTNB) which converts to the 5-thio-2-nitrobenzoate (TNB) chromophore upon reduction.

## Materials and Methods

### Materials

Sodium selenide was bought from Alfa Aesar (Bischheim, France). Metal salts, DTNB and 2-morpholinoethanesulfonic acid (MES) were from Sigma. FeCl_2_ and PbCl_2_ solutions were prepared immediately before use. Sodium selenide concentrations in solution were measured using the colorimetric assay described for hydrogen sulfide [17], [19].

### Strain and Media

The *S. cerevisiae* strain DTY7 (*MAT*α *ura3-52 leu2-3*,*112 his6 CUP1* ^R−3^) was kindly provided by Dr D. J. Thiele (University of Michigan Medical School, USA). Rich YT medium contained 1% yeast extract (Difco), 1% Bacto-Tryptone (Difco) and 2% glucose. Synthetic dextrose (SD) minimal medium contained 0.67% yeast nitrogen base (Difco), 2% glucose and 50 µg/liter of each histidine, leucine and uracil. This medium was buffered at pH 6.0 by the addition of 50 mM MES-NaOH.

### Toxicity Assays

*S. cerevisiae* strain DTY7 was pre-grown overnight at 30°C in SD minimal medium. Cells were then diluted in the same medium to get an optical density (OD) of 0.03 at 650 nm and left to grow at 30°C under agitation. When the OD_650_ reached 0.2–0.6, cells were harvested by centrifugation (10 min, 20,000 g), washed three times with 10 ml of MES buffer (50 mM, pH 6.0), and resuspended in MES buffer at a final OD_650_ of 0.2. Microtubes containing 1 ml of MES buffer with either sodium selenide at twice the desired final concentration (selenide toxicity assays) or sodium selenide plus metals at twice the desired final concentrations (protection by metals in selenide toxicity assays) were prepared in an anaerobic glove box. After incubation for 10 min (60 min in the case of iron), these tubes were closed to prevent oxygenation, taken outside the glove box, and 0.5 ml of their content was added to another tube containing 0.5 ml of the cell suspension. Before mixing, all tubes were preheated at 30°C. Since half of the mixture came from an anaerobic atmosphere, final dioxygen concentration was 117 µM instead of 234 µM if the experiment had been performed under aerobic conditions. The resulting mixture was agitated in a water bath at 30°C for 5 min before 1000-fold dilution in water. Finally, a 200 µl sample of this dilution was plated onto rich YT agar plates to monitor cell survival. Plates were left to grow for 2 days at 30°C prior to scoring.

### Monitoring of the Formation of Metal-selenide Complexes

Formation of colloidal metal-selenide complexes was monitored by measuring the changes in optical density due to turbidity. This method has already been successfully used in a selenide assay based on colloidal lead selenide formation [20]. Optical densities at 340 nm were measured inside an anaerobic glove box, with a Beckman DU 530 spectrophotometer.

### Stability of Metal-selenide Complexes in the Presence of DTNB

Attack of some metal selenide complexes with DTNB resulted in the production of yellow-colored TNB and colloidal elemental selenium (Se^(0)^). The kinetics of the reactions were followed at 412 nm in an anaerobic glove box. Turbidities of metal-selenide and elemental selenium colloids also contributed to the OD_412_ value. Maximum contributions of the metal-containing colloids were measurable before the addition of DTNB. These contributions vanish upon full complex decomposition. Upon dissolution of 1 mol of metal-selenide complex, 1 mol of elemental selenium (apparent molar turbidity coefficient of 1,300 at 412 nm) and 2 mol of TNB (molar absorbance coefficient of 14,150 at 412 nm) are produced at a same time. Therefore, along the whole kinetics, the contribution of Se^(0)^ to the OD_412_ value remains 22-times smaller than that of TNB.

We observed that three metal ions (Ag^+^, Cu^2+^ and Hg^2+^) quenched the absorbance of TNB at 412 nm, presumably through the formation of high-affinity metal-TNB complexes, thereby impairing easy detection of TNB at this wavelength. On the other hand, we observed that the copper-, mercury- and silver-TNB complexes displayed molar absorbance coefficients at 390 nm (4,800, 6,000 and 8,000, respectively) sufficiently high with respect to that of DTNB (2,400) to detect TNB formation despite the presence of the metal ion quenchers. We used this property to follow TNB formation with Ag^+^, Cu^2+^ and Hg^2+^.

### Measurement of Dissolved Dioxygen Concentration

To follow consumption of dioxygen upon catalysis by metals of the oxidation of selenide, dissolved dioxygen concentration was monitored at 30°C with a Presens Microx TX3 oximeter equipped with a needle-type microsensor (NTH-PSt1). Time needed for this sensor to reach 90% of the final output (t_90_) was 1 s. Measurements were performed in small vials (1.6 ml) open to ambient air. All solutions were buffered with 50 mM MES (pH 6.0). The experiment was started by adding 0.32 ml of an anaerobically prepared solution of selenide (or of a selenide-metal mixture) to 1.28 ml of an oxygenated solution (234 µM dioxygen) containing the metal under study. Along the experiment, the solution was mixed with a small stirring bar.

## Results and Discussion

### Mixing Various Metal Salts with Sodium Selenide Results in the Formation of Colloids

**Figure 1:**
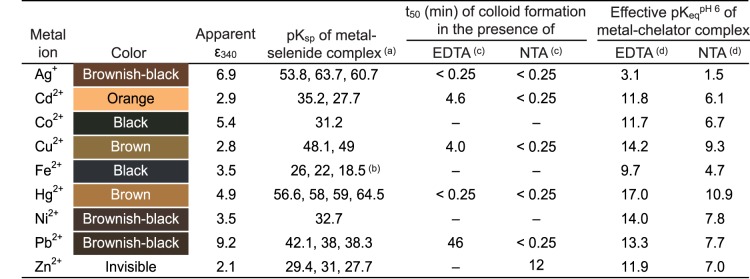
Colors and apparent molar turbidity coefficients of metal-selenide colloids. Indicated colors are those of samples containing 100 µM sodium selenide and 200 µM of each studied metal ion and incubated for 10 min. Apparent molar turbidity coefficients (ε_340_) were deduced from the data in Fig. 2, assuming that, when the metal is in excess, all the selenium has passed from the soluble phase to the colloidal phase. Under our conditions, zinc selenide colloid formation could be evidenced at 340 nm (Fig. 2) but remained invisible to the eye. Nevertheless, after centrifugation, a light yellow precipitate could be recovered. ^(a)^ pK_sp_ values of the metal selenide complexes, taken from Séby *et al.* [12], correspond to the equilibriums M^2+^+Se^2–^ ⇔ MSe (divalent cations) or 2 Ag^+^+Se^2–^ ⇔ Ag_2_Se (silver ion). Of note, in many cases, different pK_sp_ values are available in the literature for a same complex. ^(b)^ In the case of Fe^2+^, the pK_sp_ value that we determined in this study from data in Fig. 2 is also shown. ^(c)^ Sodium selenide (50 µM) was added to a mixture of metal ion (100 µM) and of either EDTA or NTA (1 mM). The sample was incubated anaerobically in a 50 mM MES buffer (pH 6.0) for 3 h. Metals for which colloid formation was no longer observed in the presence of the chelator are labeled with “**–**” (minus). For the metals which still produced colloids, t_50_ was measured. ^(d)^ Effective equilibrium constants of the chelator-divalent metal complexes at pH 6.0 (K_eq_^pH 6^) [64] were calculated using absolute equilibrium constants of these complexes (M.EDTA^2−^ or M.NTA^−^) and protonation pK values of 0.0, 1.5, 2.0, 2.68, 6.11 and 10.17 for EDTA and of 0.8, 1.8, 2.48 and 9.65 for NTA [65]. In the case of the monovalent Ag^+^ ion, effective pK_eq_^pH 6^ values were drawn from equilibrium constants of the Ag.EDTA^3−^, Ag.H-EDTA^2−^ and Ag.NTA^2−^ complexes [65].

The ability of sodium selenide to react with metal ions to give insoluble complexes was questioned. All the metal ions used in this study (Ag^+^, Ca^2+^, Cd^2+^, Co^2+^, Cu^2+^, Fe^2+^, Hg^2+^, Mg^2+^, Mn^2+^, Ni^2+^, Pb^2+^ or Zn^2+^) were given as chloride salts, apart from silver for which a sulfate salt was used due to the extremely low solubility of the chloride salt. Formation of colloidal precipitates of selenide-metal complexes was monitored through the variation of optical density due to turbidity. The OD_340_ of a mixture of 50 µM sodium selenide with 10 to 200 µM metal salts was followed over time. This range of concentrations encompassed those used in the toxicity experiments with yeast cells which are described further below. Measurements were performed inside a glove box in a nitrogen atmosphere to prevent oxidation by dioxygen of hydrogen selenide to insoluble elemental selenium, which also scatters light at 340 nm. Out of the 12 assayed metals, 9 turned into a colloid (cadmium, cobalt, copper, iron, lead, mercury, nickel, silver, zinc). Appearance of the metal-selenide colloid was immediate (less than 5 s) with cadmium, copper, lead, mercury, silver and zinc, whatever the metal concentration (10–200 µM). In the presence of cobalt, iron and nickel, the reactions with sodium selenide took a few tens of seconds and their time courses were sigmoidal. The time needed for the OD_340_ to reach half of its maximum amplitude (t_50_) was a decreasing function of added metal concentration. At 50 µM metal and 50 µM sodium selenide, t_50_ values were 17±2, 300±25 and 86±6 s with cobalt, iron and nickel, respectively (standard deviations were drawn from 3 independent experiments). Colors and apparent molar absorptions at 340 nm of the various metal-containing colloidal solutions are listed in Fig. 1.

**Figure 2:**
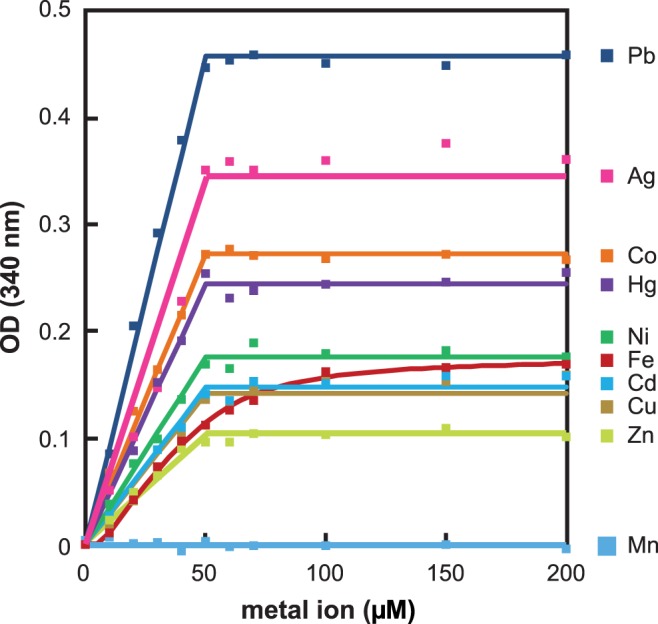
Formation of metal-selenide colloids. To monitor the formation of metal-selenide colloids, mixtures containing sodium selenide (50 µM) and the indicated metal salt concentrations were prepared inside an anaerobic glove box. After 10 min incubation at room temperature (180 min in the case of iron), the optical densities of the solution were measured at 340 nm. In the cases of cadmium, cobalt, copper, mercury, nickel, lead, silver and zinc, data were fitted to a one-site binding equation (least square minimization) assuming that the affinities between the constituents involved in complex formation were infinitely high. In the case of iron, data were fitted to the solubility product equation ([Fe^2+^][Se^2–^] = K_sp_). Obtained pK_sp_ value was 18.5±0.2. Manganese, magnesium and calcium did not modify the optical density of the sodium selenide solution. Data with manganese are shown as an example.

For 8 metal ions (Ag^+^, Cd^2+^, Co^2+^, Cu^2+^, Hg^2+^, Ni^2+^, Pb^2+^ Zn^2+^), the OD_340_ value at equilibrium increased proportionally with the added metal salt concentration and reached a plateau value for concentrations above 50 µM. Angulous shapes of the titration curves (Fig. 2) indicate strong affinity between the two constituents involved in colloid formation. Reaction products presumably are particles of metal-selenide complexes, in agreement with the low solubility products of these compounds [12]. Nevertheless, we cannot exclude that, in the case of some metals, the produced colloids also contain elemental selenium. For instance, because of the standard redox potentials of the HSe^−/^Se^(0)^ redox couple (−0.23 V) [12] and of the Cu^2+^/Cu^+^ couple (0.15 V), HSe^–^ may reduce Cu^2+^ in Cu^+^ and, therefore, the following reactions may have simultaneously occurred:
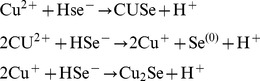



As a consequence, the precipitate might be a mixture of CuSe, Cu_2_Se and Se^(0)^.

Since sodium selenide concentration in our experiments was 50 µM, appearance of a plateau at 50 µM metal strongly suggests equimolarity between selenium and metal atoms in the insoluble complexes (Fig. 2). For silver, the concentration displayed in abscissa of Fig. 2 is that of Ag_2_SO_4_. Thus, the concentration of the silver ion in the solution is twice the indicated concentration, and the metal-selenium stoichiometry at the plateau is 2∶1 for this monovalent cation. The 8 metals readily producing colloids (cadmium, cobalt, copper, lead, mercury, nickel, silver, zinc) will now be referred to as “interacting metals”.

When Fe^2+^ (10–200 µM) was mixed with 50 µM sodium selenide, a smooth titration curve (OD_340_ values at equilibrium as a function of iron concentration) was observed (Fig. 2). This titration could be satisfactorily fitted using established protonation pK_1_ and pK_2_ values of H_2_Se and HSe^–^ of 3.9 and 15.0, respectively [12], and a solubility product constant, K_sp_ of 10^−18.5^ for the iron(II)-selenide complex.

Upon calcium, magnesium or manganese addition, the OD_340_ value of the 50 µM sodium selenide solution stayed continuously null. The results with manganese are displayed in Fig. 2. Therefore, we suspected that these metals did not produce complex with selenide in the concentration range we explored. To prove that, we performed competition experiments between Fe^2+^ and the three metals (Mg^2+^, Mn^2+^ or Ca^2+^) in complex formation with selenide. Indeed, because of the relatively low pK_sp_ value of the iron-selenide complex, we expected to easily challenge the reaction of 50 µM Fe^2+^ and 50 µM selenide by the presence of a metal competitor. Sodium selenide (50 µM) was first mixed with either Mg^2+^, Mn^2+^ or Ca^2+^ in large excess (10 mM). At this stage, the solutions remained devoid of absorbancy, as observed above at lower concentrations of these metals. Next, 50 µM FeCl_2_ was added to the three solutions as well as to a control solution containing sodium selenide only. In all cases including the control, colloidal suspensions appeared with the same kinetics (t_50_ of 5±0.5 min, data not shown). Final OD_340_ values were the same for all solutions within a confidence level of 90%. This indicated that the Mg^2+^, Mn^2+^ or Ca^2+^ metal ions, although present at a 10 mM concentration, did not interfere with the formation of the iron-selenide complex.

The behaviors of the 12 metals examined here are consistent with their known pK_sp_ values for metal-selenide colloid formation [12], as reported in Fig. 1. In particular, the pK_sp_’s of calcium, magnesium and manganese selenides are much weaker than those of the 8 above “interacting metals”. With FeSe, pK_sp_ values of 26 or 22 were published [12]. According to these values obtained on a theoretical ground, an angulous shape should have been observed for our titration curve with iron in Fig. 2. Instead, this curve indicates a pK_sp_ of 18.5±0.2. Such a discrepancy between our experimental pK_sp_ and those available in the literature possibly originates from the formation of soluble small-sized metal-selenide complexes similar to those described in the case of iron sulfides [21]. By neglecting the formation of such soluble complexes, which would not diffuse light, we may have overestimated the free Fe^2+^ ion concentration in the fitting of our titration curve to the solubility product equation.

In a second round of experiments, we challenged the formation of the metal-selenide colloids by prior addition of 1 mM EDTA to the metal ion solution (50 mM MES, pH 6.0, 100 µM metal). Upon addition of 50 µM selenide to the mixture, a precipitate was still obtained with Ag^+^, Cd^2+^, Cu^2+^, Hg^2+^ or Pb^2+^ (Fig. 1). This shows that the free energy recovered upon colloid formation with selenide is strong enough to displace these 5 metals from their complexes with EDTA. With Co^2+^, Fe^2+^, Ni^2+^ and Zn^2+^, formation of colloidal precipitates was no longer observed in the presence of EDTA. Under our experimental conditions (50 µM selenide, 100 µM of a divalent metal ion and 1 mM EDTA, pH 6.0) and taking the above pK_1_ and pK_2_ protonation values of H_2_Se and HSe^–^, application of mass action law predicts the formation of a colloid at equilibrium if pK_eq_^pH 6^+14.3<pK_sp_, where K_eq_^pH 6^ is the effective equilibrium constant of an EDTA-metal complex at pH 6.0 and K_sp_ is the corresponding metal-selenide solubility product constant. From the constants in Fig. 1, colloid formation should happen for all divalent metals but Fe^2+^. The prediction with Fe^2+^ was made using the experimental pK_sp_ value of 18.5 drawn from Fig. 2. A possible explanation to the unexpected behavior of Co^2+^, Ni^2+^ and Zn^2+^ is that, although colloid formation is predictable with these metals at infinite time, their displacement from EDTA complexes is too slow to be detected in the time range of the experiments (3 h, Fig. 1). In favor of a slowing down of colloid formation by EDTA, we observed that the times of precipitation of Cd^2+^, Cu^2+^ and Pb^2+^ complexes were shifted from seconds to minutes in the presence of the chelator (Fig. 1). To further examine this idea, we used nitrilotriacetic acid (NTA). Because this chelator binds metals more weakly than EDTA (Fig. 1), we expected that selenide would subtract metals from their NTA chelates faster than from their EDTA chelates. With NTA as the challenger of colloid formation, application of the rule pK_eq_^pH 6^+14.3<pK_sp_ again predicts colloid formation with all divalent metals but Fe^2+^. Actually, Co^2+^ and Ni^2+^ again escaped the rule. In contrast, Zn^2+^ was now slowly displaced from its NTA complex to give colloid (t_50_ of 12±2 min). This behavior possibly reflects higher kinetical dissociation constant of this cation from its NTA complex as compared to its EDTA complex.

### Insoluble Metal Selenide Complexes are not Toxic Towards *S. cerevisiae*

We then designed a viability assay to test the alleviation by added metals of sodium selenide toxicity towards yeast. Mixtures of sodium selenide with the metal salts in slight excess were anaerobically prepared inside a glove box. Tubes were taken off the glove box, and 0.5 ml of the mixtures was added to a same volume of a suspension of *S. cerevisiae* cells to obtain final concentrations of 50 µM selenide and 60 µM metal. After 5 min incubation at 30°C, aliquots of the samples were plated on rich medium. Ability to form colonies was used as an indicator of survival. Calibration experiments in the absence of metal indicated that exposure of *S. cerevisiae* cells to 5 and 10 µM selenide led to 48±19% and 98±0.5% mortality, respectively.

**Figure 3:**
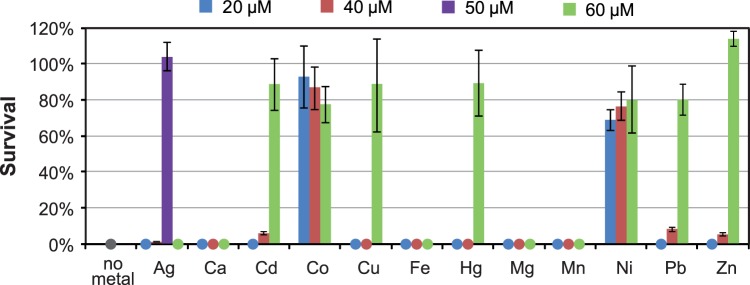
Effect of metals on the toxicity of 50 µM sodium selenide. Before plating on rich medium, DTY7 cells were exposed for 5 min to mixtures containing 50 µM sodium selenide and varying concentrations of the indicated metal salts. Colonies were counted after two days of growth. Shown in ordinate are the percentages of survival relative to controls performed in the absence of selenide and metal. Data of the histogram were obtained with 20 µM (blue), 40 µM (dark red), 50 µM (violet) and 60 µM (green) metal. Control experiment with no metal is in grey. Range bars at the top of the histogram bars were deduced from duplicate experiments.

Toxicity of 50 µM selenide was not modified by the presence of 60 µM Ca^2+^, Mg^2+^ or Mn^2+^. Notably, these metal ions do not form colloids with sodium selenide in solution (Fig. 3). We repeated the experiment with 5 µM sodium selenide, a condition which kills only ∼50% of the cells. Whatever the metal, its addition at 60 µM or at 10 mM did not modify cell death rate. In parallel, we verified the innocuousness of the 3 metals added alone. We conclude that, even added up to 10 mM, neither Ca^2+^, Mg^2+^ nor Mn^2+^ ions significantly change selenide toxicity.

When Cd^2+^, Co^2+^, Cu^2+^, Ni^2+^, Pb^2+^ or Zn^2+^ ions were present at 60 µM, the toxicity of 50 µM selenide was almost completely abolished, thereby showing that insoluble colloids with these metals were not toxic towards *S. cerevisiae* (Fig. 3). We also verified that each of these ions added alone at 60 µM was not toxic.

Because exposure to mercuric chloride is harmful [22], [23], we first evaluated the toxic effects of 60 µM and 10 µM mercury. Five min exposure to 60 µM mercury alone resulted in complete cell death. When 10 µM mercury was used, nearly 100% of the cells survived. Upon addition of 50 µM sodium selenide, the lethal effect of 60 µM mercury was suppressed (Fig. 3), thereby showing mutual detoxifying effect of selenide and this metal.

No survival was observed after exposure to 60 µM Ag_2_SO_4_ plus 50 µM sodium selenide (Fig. 3). The most likely explanation is that this condition produced 50 µM Ag_2_Se colloid but entailed enough free silver ions (20 µM Ag^+^) to kill all cells. Indeed, toxicity of free silver cations against *S. cerevisiae* is extreme. For instance, 5 min exposure to ≥1 µM of Ag_2_SO_4_ was enough to cause 100% cell death. To determine whether the Ag_2_Se colloidal precipitate is innocuous or not, we precisely mixed 50 µM silver sulfate and 50 µM sodium selenide. According to the pK_sp_ values in Fig. 1, this condition ensures that there is an extremely low concentration of selenide or silver ions. In this condition, cells were fully protected from both selenide and silver toxicities (Fig. 3).

In the presence of 60 µM Fe^2+^and 50 µM selenide, all cells died. We checked that Fe^2+^ alone (up to 10 mM) had no effect on cell viability. Actually, considering the pK_sp_ value deduced from the data in Fig. 2 (18.5±0.2), we calculated that, at 50 µM selenide and 60 µM Fe^2+^, only ∼60% of the iron is engaged into colloid, leaving enough free selenide (14 µM) to cause full cell death. In agreement with this view, we found that in the presence of 5 µM selenide and 60 µM Fe^2+^, a condition where colloid formation is not expected to occur, ∼50% of the cells survived, as in the presence of 5 µM selenide alone. In contrast, if 10 mM Fe^2+^ was mixed with 5 µM selenide, all cells survived, in accordance with the calculation indicating that nearly all the selenide is now engaged in a colloid with the metal.

### Nickel and Cobalt Ions Catalyze the Oxidation of Hydrogen Selenide by Dioxygen

The toxicities of mixtures of 50 µM sodium selenide plus 20 or 40 µM metal ions were compared. In the cases of cadmium, copper, lead, mercury, silver and zinc, cells did not survive to exposure to such conditions (Fig. 3). This behavior reflects that ≥10 µM free selenide in excess over the metal kills the cells. In contrast, with nickel and cobalt, full protection against the toxicity of 50 µM sodium selenide was observed with 40 µM or 20 µM metal only. This viability relief at such low concentrations of nickel or cobalt cannot result from the formation of a 1∶1 complex with selenide. Possibly these two metals catalyze transformation of selenide to a non-toxic selenocompound. In fact, Ni^2+^ and Co^2+^ cations were early shown to be able to catalyze the oxidation of sulfide ions by dioxygen [24]. To know whether nickel and cobalt also drive oxidation of hydrogen selenide, we monitored dioxygen consumption in mixtures containing sodium selenide and Ni^2+^ or Co^2+^. Control experiments with the other metals (Ag^+^, Ca^2+^, Cd^2+^, Cu^2+^, Fe^2+^, Hg^2+^, Mg^2+^, Mn^2+^, Pb^2+^, Zn^2+^) were also performed.

In the absence of metal, oxidation of selenide consumes dioxygen according to the reaction [25]:
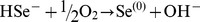
(1)


**Figure 4:**
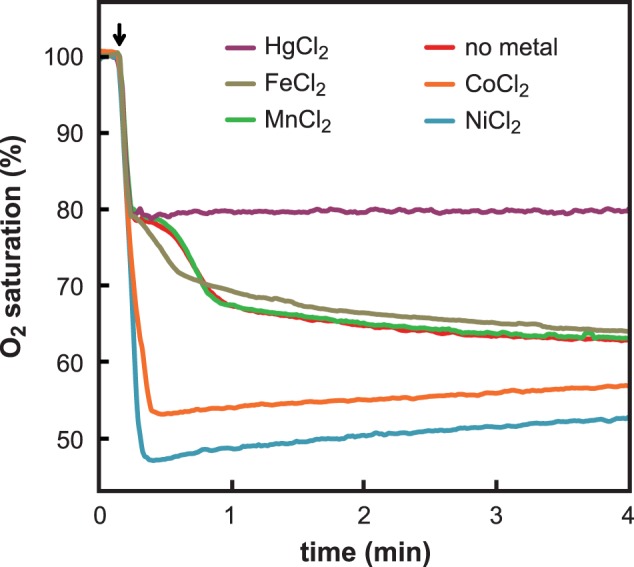
Effect of metal ions on dioxygen consumption during oxidation of sodium selenide. Dissolved dioxygen (O_2_ saturation percentage) was measured as a function of time in samples initially containing 1.28 ml of the indicated metal (125 µM). At time 10 s (as indicated by the arrow), 320 µl of an anaerobically prepared solution of sodium selenide was added to give final concentrations of 100 µM metal and 100 µM selenide. This mixing caused instantaneous drop in the dioxygen saturation level, from 100% (234 µM) to 80% (187 µM). After this drop, dissolved oxygen concentration varied depending on the assayed metal. At longer times, slight increases in dioxygen concentration were observed in the samples containing mercury, cobalt or nickel ions. These increases originate from slow diffusion of ambient air in the open vials used for the experiments. These slight increases are expected to also occur in the experiments with manganese or iron or without metal.

In our hands, this reaction spread over a few minutes (Fig. 4). The kinetics was biphasic in agreement with previous observations [25]. In the first phase, the reaction rate accelerated with time. Two non-exclusive explanations may account for this behavior. First, the auto-acceleration is reminiscent of that occurring in sulfide oxidation, where polysulfide intermediates (HS_n_^–^) might react with oxygen faster than hydrogen sulfide [26], [27]. Secondly, auto-acceleration is typical of reaction mechanisms involving free radicals [28]. Indeed, free radicals are generated during the oxidation of selenide, notably superoxide ions and hydroxyl radicals [17], [29]. The second phase, which develops as soon as elemental selenium appears (at time 1 min in Fig. 4), exhibits a classical exponential decrease.

The outcome of the addition of 100 µM metal on the kinetics of oxygen consumption by 100 µM sodium selenide varied according to the group to which the metal belonged. With Ca^2+^, Mg^2+^, Mn^2+^ and Fe^2+^, time-dependent curves of oxygen consumption exactly (Ca^2+^, Mg^2+^, Mn^2+^) or more or less (Fe^2+^) superimposed on the control recorded without metal (Fig. 4). In all cases, in agreement with equation (1), dioxygen consumed at the end of the kinetics did not exceed 50 µmol/liter. In contrast, addition of most interacting metals to the sodium selenide solution fully quenched oxygen consumption. The case of mercury is shown in Fig. 4. With nickel and cobalt, dioxygen consumption was paradoxically accelerated (Fig. 4). When the timescale of the oxidation of 100 µM selenide was minutes without metal, the reaction was completed in less than 10 s in the presence of 100 µM nickel or cobalt. Concentration of dioxygen also rapidly dropped upon addition of 20 µM of either nickel or cobalt to 100 µM sodium selenide (data not shown). This acceleration of the rate of dioxygen consumption strongly supports the idea that nickel and cobalt catalyze selenide oxidation. Dioxygen concentration also quickly decreased when 20 µM selenide and 100 µM cobalt or nickel were mixed beforehand in an anaerobic atmosphere, and then mixed with an oxygenated buffer (data not shown). This result favors the idea that pre-formed cobalt- or nickel-selenide complexes may also display catalytic properties. Fig. 4 shows that nickel or cobalt ions increased the extent of dioxygen consumption in the hydrogen selenide oxidation reaction beyond the theoretical limit value of 50 µM. Presumably, Ni^2+^- or Co^2+^-catalyzed oxidation of selenide produces elemental selenium and selenocompounds of higher oxidation state (possibly selenite or selenate). Because these oxidized forms of selenium are much less toxic than selenide towards *S. cerevisiae* [30], the rapid oxidation of hydrogen selenide which we evidence here is likely to account for the protection afforded to the cells by low concentrations of cobalt or nickel ions.

### Iron and Zinc Selenide Complexes Dissolve in the Presence of a Disulfide

**Figure 5:**
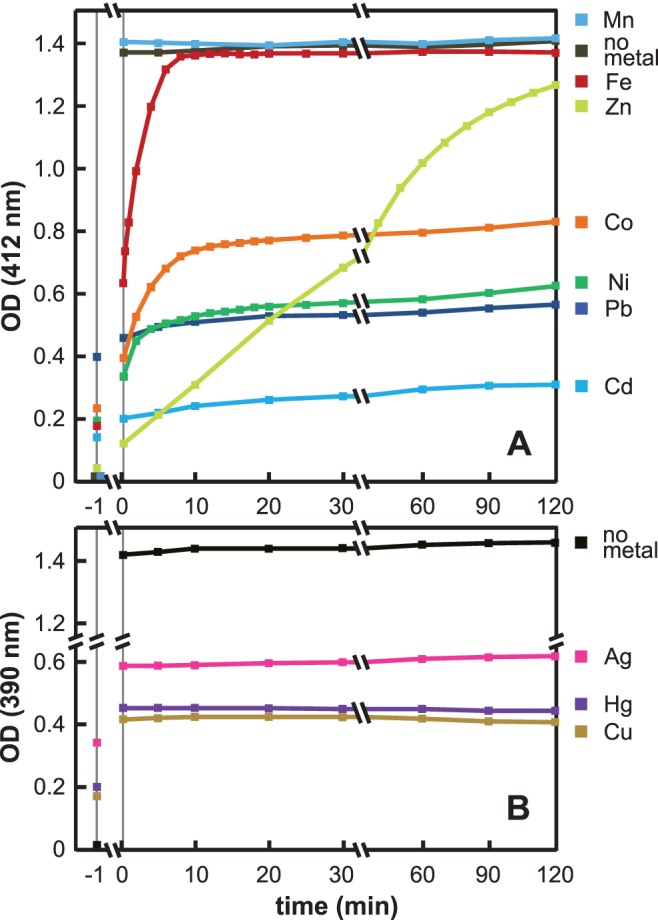
Reaction of metal-selenide colloids with DTNB. Mixtures containing 50 µM sodium selenide and 100 µM of the metal ion under study were prepared in an anaerobic glove box and left to incubate in this box for 10 min at room temperature (180 min in the case of Fe^2+^). At time zero, 100 µM DTNB was added to the samples in the glove box. (**A**) Data with Co^2+^, Cd^2+^, Fe^2+^, Mn^2+^, Ni^2+^, Pb^2+^ and Zn^2+^ ions. TNB absorbance was followed at 412 nm. Data with Ca^2+^ or Mg^2+^, which are similar to those with Mn^2+^ or without metal, are not displayed in the figure. (**B**) Data with Ag^+^, Cu^2+^ and Hg^2+^ ions. TNB absorbance was followed at 390 nm instead of 412 nm (see Materials and Methods). In all cases, turbidities of the metal-selenide mixtures were measured in the glove box before DTNB addition. Corresponding values are indicated on the left side of the figure. Next, turbidities were measured just after DTNB addition. Because DTNB slightly absorbs light at 390 and 412 nm, values measured immediately after DTNB addition (0.25 min on the figure) systematically exceeded the values before DTNB addition (−1 min on the figure). With Ag^+^, Cd^2+^, Cu^2+^, Hg^2+^, Pb^2+^ and Zn^2+^, the OD increments upon DTNB addition (0.065±0.005 and 0.24±0.01 at 412 and 390 nm, respectively) were close to those obtained in a control experiment without metal and selenide (0.060 and 0.24 at 412 and 390 nm, respectively). With Co^2+^ and Ni^2+^, the increments at 412 nm were slightly higher (0.14 and 0.16, respectively). With Fe^2+^, the increment was far higher (0.45 at 412 nm, see Results).

Because selenides are compounds prone to oxidation, we asked whether selenide-containing colloids were stable in presence of oxidizing agents. To this end, we used a disulfide (RSSR’) which is susceptible to decompose a metal-selenide (MSe) complex as follows:
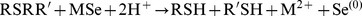



Attack of selenide in MSe is likely to occur at the surface of the colloid or through sequestration of free selenide released from the complex. We addressed the question of the stability of the various MSe complexes in the presence of a challenging concentration of a disulfide. We used DTNB, the reduction of which yields the TNB chromophore whose appearance can be monitored by optical density measurements. These experiments were performed in the absence of dioxygen. Mixtures containing 50 µM sodium selenide and 100 µM of metal salt were incubated as described in the legend to Fig. 5, before addition of 100 µM DTNB. With Ca^2+^, Mg^2+^ and Mn^2+^, an intense yellow color due to TNB formation immediately ensued. Calculation of the concentration of formed TNB indicated that nearly all of the selenide initially present in the solution had reacted. With Fe^2+^, there was a burst phase during which the OD_412_ rapidly increased, followed by a slower increase (Fig. 5). As discussed above, we believe that the iron-selenide colloid is in equilibrium with free selenide. According to the experimental pK_sp_ value which we determined above, at 50 µM total selenide and 50 µM total iron(II), this free concentration should be ∼18 µM. Consequently, the initial burst in Fig. 5 is likely to reflect reaction of DTNB with free selenide. Overall, the iron-selenide complex was dissolved by >95% after 10 min exposure to DTNB. The zinc complex reacted with DTNB much slower than the iron complex. Nevertheless, full dissolution could be reached (90% decomposition after 2 h, >95% overnight).

In the case of the nickel- and cobalt-containing complexes, a reaction with DTNB occurred. However, it remained incomplete, with the final OD_412_ corresponding to only 40–50% of the expected 100 µM TNB signal. The plateau signal did not change after overnight incubation (data not shown). The colloids in the samples remained blackish suggesting that part of the NiSe or CoSe complexes had resisted oxidation by the disulfide. Possibly, here, surface oxidation without release of formed elemental selenium limits the oxidizing reaction to the first layer of the granules. Such a surface oxidation phenomenon has already been reported in the case of NiS oxidation by dioxygen [31].

With cadmium, copper, lead, mercury and silver complexes, no decomposition by DTNB could be evidenced.

### Conclusions

In our study, we distinguished three groups of metals: (i) metals which do not protect yeast cells against sodium selenide toxicity and do not interact or weakly interact with selenide (Ca^2+^, Mg^2+^, Mn^2+^ and Fe^2+^); (ii) metals that protect cells through the formation of an extracellular insoluble complex with selenide (Ag^+^, Cd^2+^, Cu^2+^, Hg^2+^, Pb^2+^ and Zn^2+^) and, finally, (iii) metals which protect cells through either formation of a metal-selenide complex or catalysis of selenide oxidation, depending on the experimental conditions (Co^2+^ and Ni^2+^).

*In vivo*, because of chelation with various organic ligands, free heavy metal concentrations are expected to be low. Metallothioneins, for instance, display high affinity for physiological and xenobiotic heavy metals such as cadmium, zinc, copper, mercury and silver [32]. Nevertheless, we observe here that selenide can displace metals from their complexes with powerful chelators such as EDTA or NTA. Therefore, it is conceivable that metal-selenide complexes can form *in vivo* despite the presence of endogeneous chelators and despite the low free concentrations of the reactants, metal and selenium.

Indeed, various metal-selenide particles have been found *in vivo*. HgSe particles are increasingly reported to occur in the tissues of marine vertebrates and seabirds under wild conditions [6], [33], [34], [35], [36]. In the case of several marine mammals, HgSe particles were detected in liver macrophages [35], [37], [38]. Because these cells can remove nanoparticles from the bloodstream by endocytosis [39], it is imaginable that metal-Se colloid are internalized in this way. HgSe particles in liver macrophages may also originate from clearance by these cells of methylmercury-contaminated erythrocytes followed by intracellular colloid formation [38], [40]. In rats and rabbits, intravenous administration of mercury(II) and selenite produces HgSe nanoparticles which subsequently bind a plasma protein, selenoprotein P, to form (Hg-Se)_n_-selenoprotein P complexes [41], [42]. The fate of these complexes, that likely are detoxification products, is presently unknown [43].

Ag_2_Se granules were detected in the liver of several marine mammals [44]. In humans, exposure to silver led in several cases to the appearance of particles containing this metal, plus sulfur and selenium [45]. Sometimes, these particles also contained mercury, titanium and iron [46]. *In vitro* studies established that incubation of selenide with silver or cadmium plus selenoprotein P produced Ag-Se- or Cd-Se-selenoprotein P complexes similar to those obtained in the presence of mercury [47].

Very few studies were devoted to search for interactions between selenium and copper or lead *in vivo*. An association between copper and selenium has been suggested to explain the protective effect of copper on selenite and selenocystamine toxicities in rats [48]. *In vitro* addition of Pb^2+^ ions to rabbit plasma was shown to change the gel filtration pattern of selenite. The change was similar to that observed upon addition of Hg^2+^ or Cd^2+^ ions to rabbit plasma [49]. *In vivo*, formation and stability of the CuSe or PbSe complexes remain, however, to be confirmed.

Here, we show that nickel and cobalt afford yeast cell protection against extracellular sodium selenide toxicity through both complex formation and catalyzed oxidation of selenide. In biological fluids, which carry free dioxygen, the catalytic reaction could be favored. As a consequence, the occurrence of nickel- or cobalt-containing granules *in vivo* is questionable.

In the case of iron, we determined a relatively low affinity with selenide, if compared to some other metals. This may explain why the toxicity of dietary selenium in animals was not reversed by the inclusion of iron sulfate in the diet [50]. In addition, we observe dissolution of the FeSe complex by an oxidizing agent, DTNB. This behavior may also contribute to the instability of this colloid *in vivo*.

Finally, the ZnSe colloid is unstable in the presence of DTNB. This lability may explain why this complex was not detected in animals biological fluids [51]. Indeed, biological fluids contain oxidized glutathione molecules and other disulfide bonds as well as chelating molecules, which would limit or prevent accumulation of ZnSe colloidal precipitates. As we show here, prior chelation of zinc by EDTA impairs Zn-selenide complex formation.

Metals studied here include trace (Cu, Fe, Zn), ultratrace (Co, Mn, Ni and, possibly, Cd and Pb) and non-essential elements (Ag, Hg) [52]. All these metals are more or less toxic [53]. However, as we considered above, co-capture with selenide into biogenic nanospheres should afford protection. We show here which metals can be captured and how stable the resulting metal reservoirs are, depending on the redox conditions. The formation of metal-containing nanospheres also has the potential to neutralize the toxicity of selenocompounds by influencing the availability of selenium. Such a metal-dependent selenium sequestration may have consequences on these living systems which require active selenoprotein biosynthesis [54].

Several recent studies suggest that selenium is a valuable food additive to prevent some cancers [55], [56], [57], [58], [59], [60]. However, results of trials comparing the cancer incidence rate among selenium-exposed and control individuals are much debated [57], [61], [62], [63]. Variations in selenium bioavailability from one individual to another may increase the complexity background of these trials. Clearly, consumption with dietary selenium of uncontrolled levels of trace/ultratrace metals and storage of metal-selenium nanoparticles can contribute to this difficulty.
